# Evaluating the effectiveness of stepwise swallowing training on dysphagia in patients with Alzheimer’s disease: study protocol for a randomized controlled trial

**DOI:** 10.1186/s13063-022-06446-y

**Published:** 2022-06-13

**Authors:** Chenxin Wu, Kun Zhang, Junrong Ye, Xingxiao Huang, Hang Yang, Lexin Yuan, Haoyun Wang, Ting Wang, Xiaomei Zhong, Jianxiong Guo, Lin Yu, Aixiang Xiao

**Affiliations:** 1 Geriatric Psychiatry Department, Affiliated Brain Hospital of Guangzhou Medical University, 36 MingXin Road, Liwan Distrist, Guangzhou, China; 2 Nursing School, Guangzhou Medical University, 195 Dongfeng West Road, Yuexiu District, Guangzhou, China; 3 School of Public Health and Management, Guangzhou University of Chinese Medicine, 12 Airport Road, Baiyun District, Guangzhou, China; 4 Nursing Department, Affiliated Brain Hospital of Guangzhou Medical University, 36 MingXin Road, Liwan Distrist, Guangzhou, China; 5 Psychiatry Department, Affiliated Brain Hospital of Guangzhou Medical University, 36 MingXin Road, Liwan Distrist, Guangzhou, China; 6 TCM Department, Affiliated Brain Hospital of Guangzhou Medical University, 36 MingXin Road, Liwan Distrist, Guangzhou, China

**Keywords:** Swallowing rehabilitation, Alzheimer’s disease, Dysphagia, Randomized controlled trial, Protocol

## Abstract

**Background:**

The high prevalence of dysphagia among Alzheimer’s disease (AD) patients has become a public health and economic concern. Therefore, effective and accessible dysphagia treatments are needed. As a fundamental rehabilitation of dysphagia, swallowing muscle exercises have received increased attention. Stepwise swallowing training (SST), integrated with all swallowing organs movement, is expected to improve swallowing dysfunction among AD patients. By using a randomized controlled trial design, we propose a multi-center research to evaluate the effectiveness of SST program among AD patients.

**Methods:**

A multi-center exploratory randomized controlled trial, with a 4-week follow-up period, will be conducted in three major public psychiatric hospitals in Guangdong, China. Participants in the control group will be assigned to routine dysphagia care, while participants in the intervention group will undergo the same nursing care and additionally receive the SST program. The SST program includes five sections of swallowing organs training: lip movement, facial movement, tongue movement, mandibular movement, and neck movement. Primary outcomes evaluate the swallowing function, namely, Water Swallowing Test (WTS) and Standard Swallowing Assessment (SSA). Secondary outcomes aim at measuring the improvement of negative impacts of dysphagia, namely eating behavior, ability of daily activity, and nutritional status. Data will be collected at baseline (*T*_1_), at 2 weeks (*T*_2_, intervention), and 4 weeks after intervention (*T*_3_, follow-up).

**Discussion:**

This study will offer trial-based evidence of the effectiveness of SST in relieving dysphagia among AD patients. SST program is expected to improve both the swallowing function and reduce the negative impacts of dysphagia, with an exploration of acceptability in the SST program.

**Trial registration:**

Chinese Clinical Trial Registry, ChiCTR2200056481. Prospectively registered on 6 February 2022.

## Background

Alzheimer’s disease (AD) has become one of the largest global economic burdens. According to the 2015 World Alzheimer’s Disease Report, the total global cost of dementia will increase at the rate of more than 40% between 2015 and 2030—from 818 billion dollars to 2 trillion dollars [1, 2]. However, recent research suggested that China’s domestic socio-economic burden has been overwhelmingly underestimated; this will significantly affect the estimated global cost. In 2015, the cost of AD in China has reached 1.47% of the gross domestic product (GDP), while the worldwide cost accounted for 1.09% of the global GDP, indicating a higher socio-economic cost in AD patients in China. Moreover, the annual cost per AD patient in China was 19 144.36 US dollars (USD), and the annual social and economic cost of AD was 167.74 billion USD, which was 5.95 times higher than the estimated value in the aforementioned report [2]. Additionally, by 2030, a total annual cost related to dementia will reach 50.749 billion dollars [3].

The high socioeconomic burden of AD is mainly due to caregiving demands. Caring for AD patients is highly challenging for their caregivers and family members. The primary difficulty in caregiving is caused by the decline in AD patients’ daily living ability, specifically, the swallowing dysfunction. Clinically, the swallowing dysfunction leads to malnutrition, dehydration, weight loss, fear of eating, and other complications; consequently, it might extend hospital stay and even cause severe injury or death in extreme cases. Swallowing dysfunction or dysphagia, which is a group of clinical syndromes, can cause various diseases. Dysphagia refers to an improper transfer of food from the mouth to the esophagus and the stomach, caused by the impairment of the swallowing organs (e.g., mandible, lips, tongue, soft palate, larynx, esophagus, and so on). Generally, the swallowing process includes the following four physiological stages: oral preparatory, oral, pharyngeal, and esophageal [4].

Significant attention is needed to address the high prevalence of dysphagia in AD patients. Previous studies indicated an incidence rate of 32–45% among mild AD patients with dysphagia and 84–93% among moderate and severe AD patients. Abnormal swallowing function in AD patients manifested as weak tongue movement and pressure, generally occurring in the oral and pharyngeal stages of swallowing. Hence, due to abnormal swallowing function, individuals might exhibit delayed pharyngeal reflex, reduced pharyngeal muscle strength, and food residue after swallowing. Individuals with abnormal swallowing function experience difficulty in forming and pushing the food bolus and, hence, are at high risk of food aspiration [5].

Studies have reported that AD patients with dysphagia at admission are at a higher risk of malnutrition than those without dysphagia, which is associated with the respiratory infection and increased mortality [6]. When screened using the Water Swallowing Test, the incidence rate of malnutrition was 1.67 times higher, with which the severity of dysphagia was positively correlated. Besides, malnutrition also reduces the quality of life in patients [4]. A multi-center cohort study found that swallowing dysfunction was an important cause of pneumonia and lower respiratory tract infection in 170 elderly people in a nursing home (OR = 2.000, 95% CI = 1.2–3.3, *P* = 0.10) [7]. Thus, optimal solutions are urgently needed to improve patients’ swallowing function and living ability.

Dysphagia treatment includes three approaches: compensatory strategies, swallowing rehabilitation, and other approaches. The first aims to reduce the effects of impaired bolus flow to ensure the safety of oral diets [8]; it has many forms, such as postural adjustment, diet modification, swallow maneuvers, and enteral feeding. Changes in head or body posture are recommended to reduce aspiration or residue. Many postural techniques including (but not limited to) head down and lift and side-lying can successfully eliminate aspiration on at least one bolus volume of liquid [9]. Logemann et al. conducted a study using thin liquids with either chin-tuck or nectar-/honey-thickened liquids in individuals with dementia or Parkinson’s disease (PD). Overall, fewer participants aspirated on nectar- (*p*< 0.01) and honey-thickened liquids (*p*< 0.01) [10]. While previous studies are conflicted about these strategies and some data suggest that postural adjustments are inferior to active rehabilitation, other approaches include chemo-denervation, pharmacological treatment, neuromuscular electrical stimulation, and non-invasive brain stimulation [11, 12]. Chemical myotomy and drug application are not highly recommended as first-line treatment in older adults with dysphagia due to the potential risks and side effects.

Wang et al. synthesized 27 randomized controlled trials to explore the effect of noninvasive neurostimulation therapies (repetitive transcranial magnetic stimulation [rTMS], transcranial direct current stimulation [tDCS], and surface neuromuscular electrical stimulation [sNMES]), which work through magnetic or electric fields to trigger and regulate the depolarization of cortical neurons, on dysphagia patients after stroke. A positive effect of rTMS, tDCS, and sNMES was reported in the recovery of swallowing function (standardized mean difference = 0.91; 95% CI: 0.54–1.27; *Z* = 4.84; *P* < 0.00001; *I*^*2*^ = 86%). However, there is no recommended treatment protocol, and for implementation, a specific equipment is required [13].

Swallowing rehabilitation comprises exercises targeted to train specific muscles or muscle groups [8]. Given this development, more evidence-based therapeutic exercises were introduced—instead of centering on an isolated muscle, the training program gradually became more systematic and available. Kim et al. explored the instant effect of simple oral exercise (SOE), performed two times per day for a week, on 84 older adults. Masticatory performance improved immediately by around 16% in the poor-chewing group, and the unstimulated saliva production in all subjects increased to 0.26 ml/min immediately after SOE [14]. Kang et al. examined the impact of a bedside exercise program, which comprised oral, pharyngeal, laryngeal, and respiratory exercises. After implementing the program in 25 stroke patients for 1 h per day for 2 months, the results showed a significant improvement, compared to the control group, in the swallowing function (at the oral phase) and depressive symptoms [15].

However, swallowing exercises, the fundamental rehabilitation for dysphagia, need more high-quality evidence regarding their implementation in AD. Under the neural regulation mechanism of the cerebral cortex sensorimotor, practicing swallowing exercises is effective for dysphagia with stroke/PD or older adults [16, 17]. Clinicians find the implementation of appropriate rehabilitation exercises challenging in patients with AD. In China, clinicians apply swallowing exercises, such as lip lordosis and adduction, mandibular opening and closing, cheek blowing, and tongue extension, generally combined with routine care and other treatments. These exercises can be effective in increasing the flexibility and coordination of swallowing organs and improving the relevant muscles’ strength. Nonetheless, whether swallowing exercises are effective in dementia is controversial. Therefore, simple and feasible swallowing rehabilitation training should be developed for patients with impaired cognition and attention deficit. Based on the current swallowing rehabilitation literature, a multi-center randomized controlled trial will be conducted in three mainland hospitals to investigate the effect of stepwise swallowing training on the function and daily living ability of AD patients.

### Objectives

Based on the literature and clinical evidence, a program has been developed to improve AD patients’ swallowing function and muscle training from top to bottom on lips, tongue, face, and neck. The study aims to evaluate the effectiveness of the Stepwise Swallowing Training (SST) in swallowing function among AD patients with dysphagia.

## Methods

### Trial design

This training program is a multi-center, exploratory, single-blinded, parallel randomized controlled trial (RCT) with a 4-week follow-up period. All stages are conducted according to the SPIRIT reporting guidelines [18]. Treatments administered will be allocated randomly according to a 1:1 ratio by an online random-number generator of SPSS software by research assistants who are not involved in assessment and intervention. The researcher will then offer the research assistants in each hospital the allocation of groups with sequentially numbered opaque, sealed, and stapled envelopes. Then, the training schedule will be conducted based on this research protocol.

### Study settings and participants

This trial will be conducted in three major public psychiatric hospitals in Guangdong, China—Affiliated Brain Hospital of Guangzhou Medical University (GZ), the Third Hospital of Jiangmen (JM), and the Third Hospital of Yuebei (YB). This research has obtained ethical approval from the Institutional Review Board of GZ (approval number: 2021070), and the research will be conducted with the consent of each hospital’s executives. Before allocation, informed consent will be obtained from all participants.

### Inclusion and exclusion criteria

A total of 100 participants of male and female aged above 60 years with AD will be recruited from the three main research institutes. Inclusion criteria are (a) diagnosed as AD by DSM-5; (b) score ≤ 25 on the Mini-mental State Examination (MMSE); (c) score a third-degree or higher on the Water Swallowing Test, demonstrating a risk of aspiration [19]; and (d) have basic communication ability to complete the tests in this trial. Exclusion criteria are (a) swallowing disorders caused by other organic diseases, such as oral disease, esophageal obstruction, digestive tract disease, tumors, or stroke; (b) complications such as other serious somatic diseases that are not suitable for practicing swallowing exercises; and (c) serious impairment in hearing or vision, which may hinder following instructions.

### Interventions

**Fig. 1:**
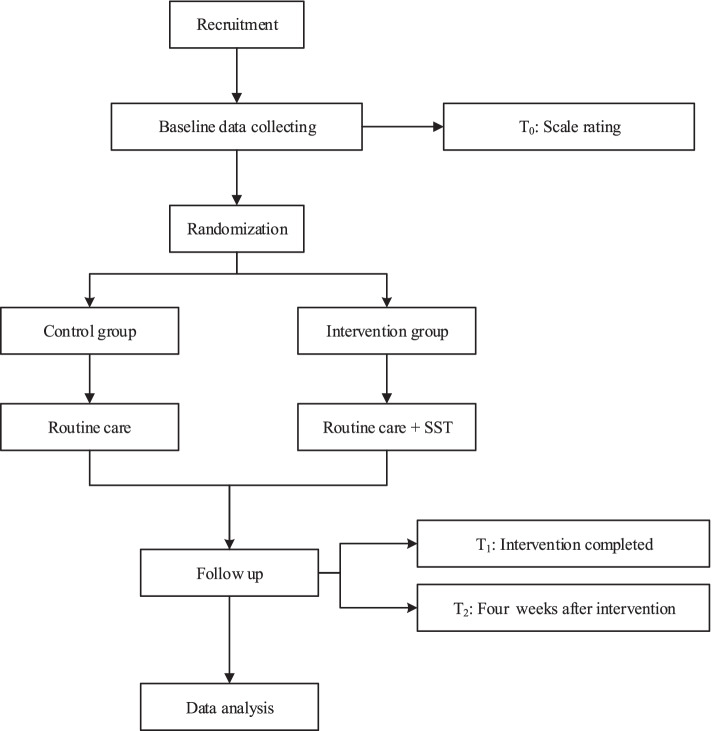
Flow chart of the trial

**Fig. 2:**
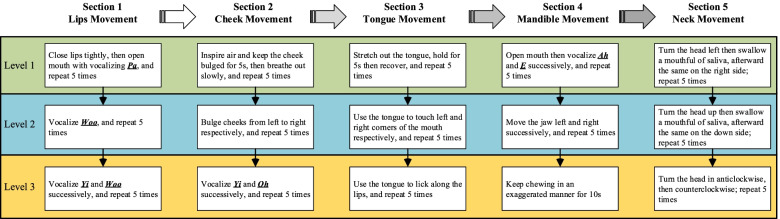
SST flow chart

Participants will be randomly allocated to either the SST group or the control group. The flow chart of this study was presented in Fig. 1*.* Participants in the control group will receive routine dysphagia care, while the intervention group will receive SST based on routine care five times a week for four weeks. The process includes five sections of swallowing muscles, and each section will provide step-by-step training, according to the difficulty experienced by the participant. Each section will be divided into three levels of difficulties, starting from level 1(easy) through level 2 to level 3 (difficulty) each time. As presented in Fig. 2, the instructional video will be shown to the patient along with one-to-one guidance.

Moreover, training videos and anime pamphlets will be given to family members and caregivers when participants are discharged from the hospital, and they will be contacted via telephone or WeChat to ensure the training process and measurements.

#### Routine nursing care during intervention

During the intervention and follow-up, participants and their caregivers in both groups will receive routine nursing care and health education about swallowing dysfunction.

#### Concomitant treatments and medication

Routine medications used to control the underlying diseases such as hypertensive and diabetic mellitus will be permitted. Extra benzodiazepines, antipsychotics, and anti-epileptics, which can affect the swallowing function can be taken if necessary. If the participants take medication that might affect their swallowing function, it will be recorded in case report form.

### Outcomes

**Fig. 3:**
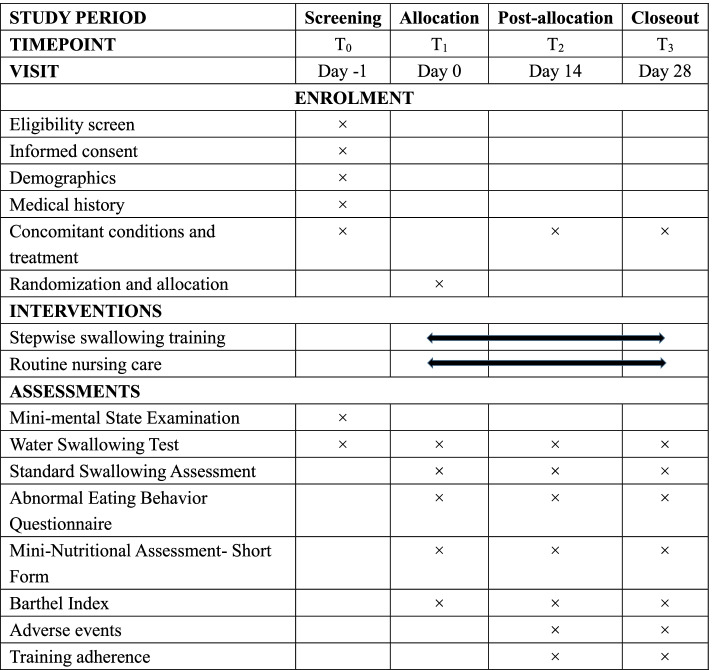
Schedule of outcome measurement

Study outcomes will be evaluated by trained research assistants who are not aware of group allocation. The time points for measurement will be at baseline (*T*_1_, day 0), 2 weeks after intervention (*T*_2_, day 14), and 4 weeks after intervention (*T*_3_, day 28). The schedule of outcome measurement is presented in Fig. 3.

#### Primary outcomes

Water Swallowing Test (WST), a five-level water drinking test, will be used to determine the effectiveness of SST. Evaluators observed individual reactions after drinking 30 ml of water (complete recovery: swallowing without cough; improved swallowing function: occasional choking, and invalid: unchanged or worse swallowing function). Effective rate = (completed recovery + improved swallowing function)/total cases in each group *100%. The sensitivity and specificity of WST ranged from 34.8 to 55.7% and 78.9 to 93.2% in older adults [20].

On basis of WST, Standard Swallowing Assessment (SSA) will be performed to evaluate the severity of dysphagia. The score of SSA ranged from 18 to 46, and a high SSA score reflected serious dysphagia. SSA exhibited a specificity of 0.90 and a sensitivity of 0.97 in detecting dysphagia, with positive and negative predictive values of 0.92 and 0.96 [21].

#### Secondary outcomes

Abnormal Eating Behavior Questionnaire (AEBQ) will be applied to investigate the frequency and the developing order of abnormal eating behavior. The 27-item AEBQ Chinese version is comprised of five domains of behaviors, namely swallowing disorders, appetite changes, food preferences, eating habits, and other abnormal eating behaviors. The single item score was calculated as frequency (ranging from 0 to 4, representing from never happen to more than once per day) * severity (ranging from 1 to 3, representing mild, moderated, and critical influence on eating behavior, respectively). The Cronbach’s *α* of frequency, severity, and the total score of AEBQ-Chinese version were 0.73, 0.74, and 0.74, respectively [22].

Mini-Nutritional Assessment-Short Form (MNA) is employed to screen malnutrition. MNA is comprised of 6 items, including weight loss, body mass index (BMI), acute diseases, mobility, mental disease, and dysphagia or anorexia. The score range of MNA is from 0 to 14, MNA<11 suggests being at risk of malnutrition, and MNA<8 indicates malnourished status. The specificity and sensitivity of MNA in predicting malnutrition were 88.8% and 85.6%, respectively [23].

The activity of daily living will be evaluated using Barthel Index (BI), a 10-item rating scale assessing individual functional capacity. The score range of BI varies from 0 to 100, and the higher BI score reflects the greater daily living activity. The empirical study yielded the test-retest reliability and interrater reliability of BI were 0.89 and 0.95, respectively [24] .

Adverse events and unexpected symptoms that happen during the intervention timeframe will be recorded and analyzed. The safety coefficient of each group is calculated as follows. Safety coefficient = (the number of adverse events/the number of engaged participants) * 100%.

The proposed study will use attendance rate to evaluate participants’ intervention adherence. Individual attendance rate = (actual number of participation/total number of intervention session) * 100%.

### Sample size

The sample size was calculated based on the comparison of WST. By using G*Power 3.1 software, we set a significant difference of 14.2% (74.2% in the experimental group and 60.0% in the control group), an alpha error of 0.05, a beta error of 0.80, and the effect size *f* = 0.32. After calculation, a total sample size of 78 was required. To accommodate for a 20% attrition rate and other confounding factors, we recruited 100 participants. After obtaining informed consent, each involved hospital was allocated to the intervention group or to the control group in a 1:1 ratio.

### Recruitment

The proposed study will recruit AD patients hospitalized in sampling sites. Water Swallowing Test, an indispensable part of the medical assessment conducted after admission, will be applied to scan the swallowing function of AD patients. Patients who meet the inclusion criteria will be invited to participate in this study. The proposed study will recruit participants in three public tertiary psychiatric hospitals, namely Guangzhou, Shaoguan, and Jiangmen City, in which the annual number of admission of AD patients were approximately 380, 200, and 320, respectively. Based on the pilot investigation, the prevalence of dysphagia among AD patients was 38.6%; thus, we estimate that the number of participants who meet inclusion criteria is (380 + 200 + 320) * 1.5 years * 38.6% ≈ 521>100. Additionally, the participants’ recruitment timeframe would be extended if necessary.

### Blinding

This is a single-blinded study. Study outcomes will be evaluated by trained research assistants who are not aware of group allocation. Participants will not be told about the pattern of research allocation. During the intervention, blinding would be unveiled if serious complications and adverse events occur.

### Data collection and management

The impacts of swallowing training will be collected at baseline (*T*_1_: allocation) and at 2 weeks (*T*_2_: intervention period) and 4 weeks after intervention (*T*_3_: closeout). Other outcomes, including adverse events, attendance rate, and attrition rate, will be continuously collected every 2 weeks from baseline. The schedule of study procedures was presented in Fig. 3. Research assistants will be trained together to ensure consistent results. After the measurement, data will be double-checked for consistency, accuracy, and integrity. Raw data will be anonymized and stored on a secured computer database. In accordance with the regulation governing research activities, data will be monitored by the Department of Research Project Administration of GZ. Research data could only be accessed by authorized members of the trial team. Anonymized trial data would be shared to enable further prospective meta-analyses after having the permission of the project investigator (AX).

### Statistical methods

Exploratory data analysis and Shapiro-Wilk tests will be performed to determine the normality of data distribution. Continuous variables will be expressed as means with standard deviations or medians with interquartile ranges; mean differences will be expressed from baseline to the end of the study. For categorical variables, counts and percentages will be presented, and between-group comparisons at baseline will be tested with the *x*^*2*^ test [25]. Between-group differences at baseline, and in the change from baseline to the end, will be tested with repeated measure analysis of variance. An analysis of covariance will be used to adjust for baseline cognitive functioning. Paired *t*-tests will be performed for between-group comparisons from the baseline to the end. The level of significance will be set as a two-sided *P* value less than 0.05. Data analysis will be conducted using SPSS version 20.0 with intent-to-treat analysis (ITT) and per-protocol (PP) analysis. The results of the ITT analysis will be compared with those of the PP analysis to determine the consistency.

### Study quality control

The validity and reliability of the instruments used in this study have been examined. The psychiatric hospitals involved in this study are qualified in research and geriatric mental care and can offer adequate support. To improve homogeneity, all research assistants will receive unified training for proper assessments and check the questionnaire; the intervention will use standardized teaching videos and instruction. Moreover, the primary researcher will check the videos recorded during the intervention twice weekly for the performance adherence of patients and the staff.

## Discussion

This protocol presents a multi-center and exploratory RCT to assess the effectiveness of progressive swallowing exercises on swallowing function, daily activity life, and eating behaviors in AD patients in Guangdong, China. The intervention program addresses five main swallowing muscle groups and will be taught stepwise.

### Monitoring

Adverse events, data collection, maintenance of data confidentiality, and data analysis will be monitored by the Department of Research Project Administration of GZ. A research assistant will be assigned to each hospital to conduct a biweekly review of the study progress and any adverse events of this study. Any unexpected event will be directly reported to the Department of Research Project Administration of GZ.

### Risks, burdens, and benefits

The intervention will be implemented based on the patient’s physical condition. The physical and emotional distress induced by the intervention (e.g., an increase in respiratory and heart rates) are assumed to fall within the range of a normal reaction. To reduce the discomfort during SST, research assistants will maintain participants’ hydration and ensure appropriate rest. In a previous study, the Water Swallowing Test score and Barthel Index improved after intervention [26]. This study has been assessed by the ethical department in Guangzhou mental health center as a non-invasive program. Based on these assessments, the training program designed for dysphagia is not harmful to AD patients.

Before commencement, to avoid the risk of medical emergencies during the implementation, the nurses and research assistants will be instructed about the safety considerations and safety protocol. The caregivers or family members will respond to the questionnaire, while the participants will undergo the cognitive function test, that is, MMSE. Participation in the study will provide no direct benefit to the respondents.

### Modification and inquiry

Any modification or inquiry regarding the study will be discussed through the Affiliated Brain Hospital of Guangzhou Medical University. During the study period, inquiries from the patients and their families will be gathered and solved by research assistants.

### Confidentiality

The participants’ personal data, including names, addresses, and telephone numbers, contained in the consent form and case report form will be anonymized using a research ID number and stored on a secured computer database. Research data will be monitored by the Department of Research Project Administration of GZ and will only be accessed by authorized members of the trial team.

### Post-trial care

If the study demonstrates no negative effects, participants in the control groups will be invited to participate in SST program after the study timeframe.

### Dissemination policy

The results will be disseminated to professionals and hospitals in geriatric and mental care in Guangdong province. The findings will also be disseminated to peer-reviewed scientific journals and presented at academic conferences.

## Trial status

This manuscript describes the SST trial protocol version #1 dated February 6, 2022. Recruitment started in March 2022 and the anticipated completion is March 2023.

## Data Availability

The datasets will be available from the corresponding author on reasonable request.

## Abbreviations

GDP: Gross domestic product
SST: Stepwise Swallowing Training
RCT: Randomized controlled trials
MMSE: Mini-Mental State Examination
WST: Water Swallowing Test
SSA: Standardized Swallowing Assessment
AEBQ: Abnormal Eating Behavior Questionnaire
MNA-SF: Mini Nutritional Assessment - Short Form
VFSS: Video Fluoroscopic Swallowing Study
BI: Barthel Index
ITT: Intention-to-treat
PP: Per-protocol
